# RSV-specific humoral and cellular immune responses in kidney transplant recipients

**DOI:** 10.3389/ti.2026.17558

**Published:** 2026-10-30

**Authors:** Britte G. Lenderink, Laura L. A. van Dijk, Marjolein Dieterich, Marcia M. L. Kho, Sebastiaan Heidt, Rory D. de Vries

**Affiliations:** 1 Department of Internal Medicine, Erasmus MC Transplant Institute, Erasmus Medical Center, Rotterdam, Netherlands; 2 Department of Viroscience, Erasmus Medical Center, Rotterdam, Netherlands

**Keywords:** cytokines, kidney transplant recipients, neutralizing antibodies, respiratory syncytial virus (RSV), T cells

## Abstract

Induction of virus-specific immune responses in kidney transplant recipients (KTR) by vaccination is challenging. However, it is not well-described whether pre-existing immunity against respiratory viruses initially encountered before transplantation, like respiratory syncytial virus (RSV), is maintained. Therefore, we assessed RSV-specific humoral and cellular immune responses in KTR and controls at two timepoints during the COVID-19 pandemic. Binding and neutralizing antibodies were determined in serum samples from 64 KTR and 37 controls. Cellular immune responses were measured in a cohort subset (15 KTR, 14 controls) by stimulation of peripheral blood mononuclear cells with RSV-derived peptide pools. This was followed by phenotypic analysis using an activation-induced marker assay and cytokine release assay. RSV-specific binding IgG and neutralizing antibody titers were comparable between KTR and controls and stable over time. While total lymphocyte levels were significantly lower in KTR, relative frequencies of RSV-specific CD4^+^ and CD8^+^ memory T cells were comparable between groups. Unsupervised hierarchical clustering distinguished three cytokine production profiles with divergent RSV-specific immune responses, but KTR and controls were equally distributed throughout. Thus, KTR have comparable RSV-specific humoral and cellular immune responses to controls. Our findings suggest that pre-existing respiratory virus-specific immune responses persist under immunosuppressive treatment.

## Graphical Abstract

**Figure:**
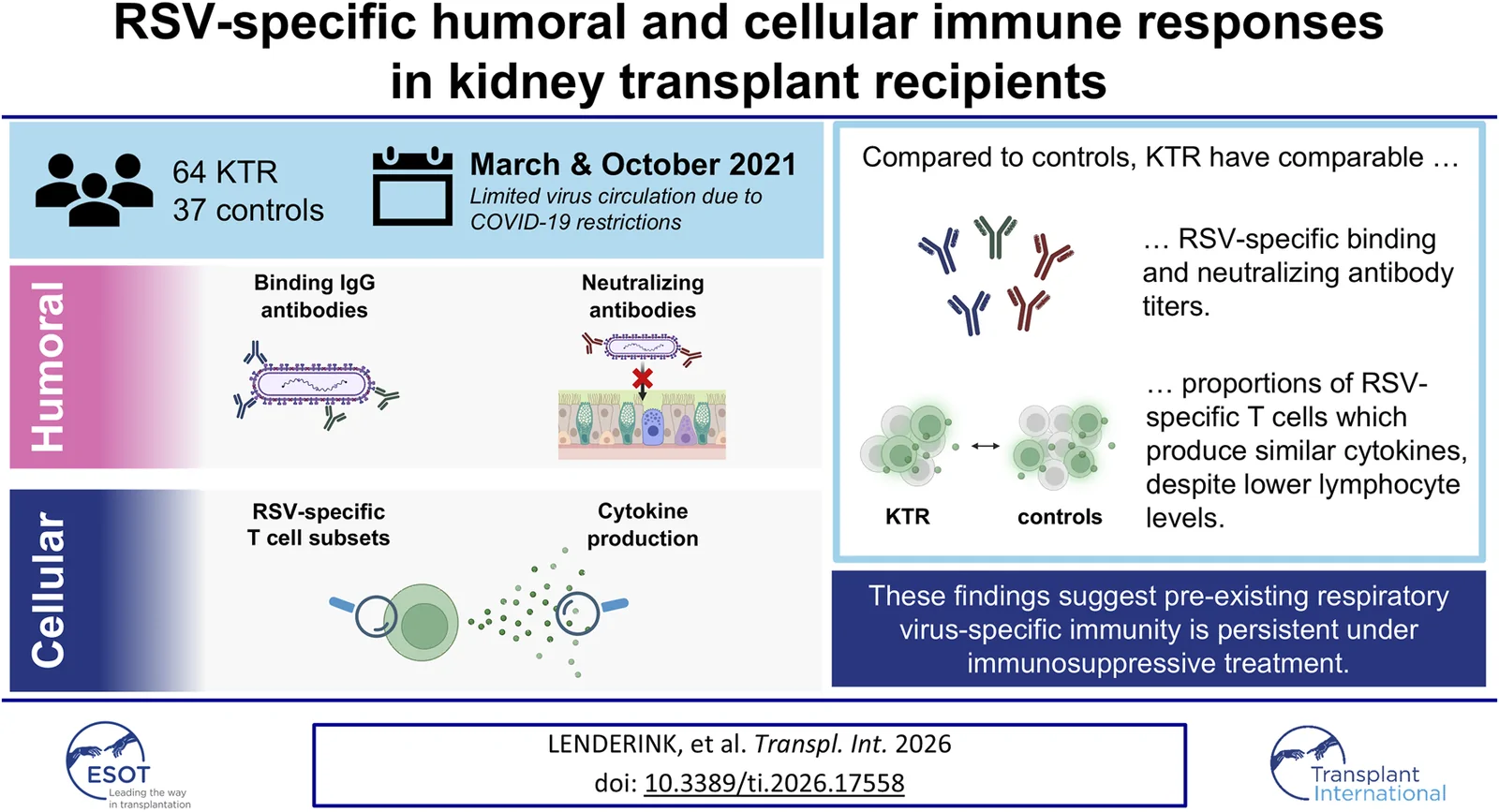

## Introduction

Transplantation is the superior treatment option for patients with end-stage kidney disease. In the past thirty years, patient and graft survival have greatly improved in part due to improved immunosuppressants [1]. While beneficial for graft survival, immunosuppressive regimens make kidney transplant recipients (KTR) vulnerable to developing severe disease after respiratory viral infections [2–4]. Additionally, *de novo* induction of virus-specific immune responses is challenging in this patient population, as demonstrated by the need for multiple COVID-19 vaccinations to reach protective antibody levels [5–7]. It is generally assumed that pre-existing immunity against respiratory viruses initially encountered during infancy (and therefore prior to transplantation), such as respiratory syncytial virus (RSV), is maintained after transplantation, but this is not well-characterized in KTR.

RSV mainly causes severe disease in infants, the elderly and immunocompromised individuals [8]. It is a member of the *Pneumoviridae* genus with a negative sense RNA genome encoding for 11 proteins, among which the surface fusion (F) and attachment (G) proteins [8]. RSV is classified into two genetically distinct subgroups, RSV-A and RSV-B, with the greatest variability observed in the G protein [9, 10]. The G protein interacts with the CX3CR1 receptor on airway epithelial cells, after which the F protein undergoes conformational changes, thereby mediating fusion between the viral particle and the target cell [11]. The F protein is metastable and exists in a fragile pre-fusion (pre-F) and a highly-stable post-fusion conformation; predominantly antibodies specifically targeting the pre-F conformation determine the magnitude of neutralizing activity in human serum [12]. Consequently, the majority of prophylactic monoclonal antibodies in clinical use target epitopes in the pre-F conformation, and EMA-approved RSV vaccines consist of the pre-F stabilized protein [13].

To better understand the magnitude and phenotype of RSV-specific immune responses in KTR in relation to controls, we measured humoral and cellular immune responses in paired samples obtained in March and October 2021. Due to COVID-19 restrictions, circulation of respiratory viruses was limited from March 2020 to May 2021 in the Netherlands, thus permitting a baseline perspective of RSV-specific immune responses.

## Materials and methods

### Participants

For this study, we used samples obtained in the context of a prospective, open-label, randomized, controlled trial as part of the Immune Response study of the RECOVAC (Dutch REnal patients COVID-19 VACcination) consortium, described elsewhere [6]. The studies involving human participants were reviewed and approved by the Erasmus MC ethics committee. The participants provided written informed consent to participate in the study. Ethical approval for our study was obtained from the Erasmus MC ethics committee (MEC-2025-0115). Serum and peripheral blood mononuclear cells (PBMCs) were collected in March and October 2021. Selection of participants for our study was based on availability of materials, which resulted in the inclusion of 64 KTR and 37 individuals without kidney disease (controls). For the PBMC analysis, we randomly selected 15 KTR and 14 controls with PBMCs available at both timepoints.

### Humoral RSV-specific immune responses

To determine humoral RSV-specific immune responses, we measured binding IgG and neutralizing antibody titers. Binding antibody titers were measured against the G protein of RSV-A (Ga) and pre-F protein using enzyme-linked immunosorbent assay (ELISA). Briefly, 96-well high binding plates (Corning Incorporated, ME, USA) were coated with 100 ng stabilized pre-F (DS-Cav1, NIH, USA) or Ga (RSV A, rsb1734, Sino Biological, Beijing, China) protein in phosphate-buffered saline (PBS, in-house) per well and incubated overnight at 4 °C or for 1 h at 37 °C. The plates were then blocked for 1 h at 37 °C with 100 µL blocking buffer (Blocker^TM^ BLOTTO in tris-buffered saline (TBS, Thermo Scientific, MA, USA) diluted 1:1 in PBS). After blocking, sera were added in 3-fold dilutions in blocking buffer starting at 1:40 (50 µL/well) and incubated for 1 h at 37 °C. This was followed by adding 1:6,000 polyclonal rabbit anti-human IgG/HRP (DAKO, CA, USA) (50 µL/well) and incubation for 1 h at 37 °C. Subsequently, KPL TMB peroxidase substrate diluted 1:1 with TMB diluent (SeraCare, MA, USA) was added and incubated for 10 min at room temperature (RT). Finally, the reaction was stopped by adding 100 µL sulphuric acid and plates were read out at 450 nm with 620 nm background in a plate reader (Tecan, Männedorf, Switzerland). The absorbance values were used to determine the 50% inhibitory concentration (IC50), considered the binding antibody titer, by fitting a log-logistic function on the S-curves using the R package drc (v3.0-1).

Recombinant RSV containing EGFP as an additional transcription unit [rHRSV^A11^EGFP(5)] was kindly gifted by Dr. W.P. Duprex [14]. Virus stocks were generated in and titrated on HEp-2 cells as previously described [15]. Titers were expressed in tissue culture infectious dose-50 (TCID50)/mL as calculated using the Reed & Muench method [16]. Neutralizing antibody titers were determined by virus neutralization (VN): in brief, a 2 or 4-fold dilution series, starting at a 1:32 serum dilution, was incubated in duplicate with ∼1,000 TCID50 rHRSV^A11^EGFP(5). This virus-serum mixture was then added to 4–5-day old monolayers of Vero cells and incubated for 2 days. Plates were scanned on a Typhoon laser-scanning platform (Cytiva Life Sciences, DE, USA) and fluorescent foci were counted using ImageQuant TL v8.2.0.0. Counts were used to determine the 50% plaque reduction neutralization titer (PRNT50), considered the neutralizing antibody titer, by calculating the proportionate distance as described previously [17].

### Cellular RSV-specific immune responses

RSV-specific cellular immune responses were detected by stimulation of PBMCs with RSV-derived peptide pools. First, PBMCs were thawed and rested for approximately 6 h in a 96-well plate (Greiner, Frickenhausen, Germany). Cells (1 million/well) were subsequently stimulated for 21 h with 1 μg/mL RSV peptide pools (Supplementary Table 1, JPT Peptide Technologies, Berlin, Germany). These peptide pools consist of 15-mer peptides with an 11-amino acid overlap encompassing the surface proteins F from both RSV strains (Fa, Fb) or Ga, the internal proteins M or N, or a selection of known immunogenic peptides (Select). The solvent DMSO (0.2%, VWR international, Fortenay-sous-Bois, France) served as negative control, whereas Concanavalin A (1:200, ConA, Sigma-Aldrich, MO, USA) served as positive control. Stimulations with DMSO, ConA and Select were always performed. Cell availability determined the number of stimulations that could be included per sample: for each additional 1 million cells available, an extra stimulation was included in the following order: Fa, Ga, Fb, N and M. After PBMC stimulation, we determined the relative frequency of RSV-specific T cells and measured cytokine production in the supernatant.

To assess the relative frequency of RSV-specific T cells, we performed a flow cytometry-based activation induced marker (AIM) assay. Flow cytometry staining was performed by incubating cells with a Live/Dead marker for 15 min at RT in the dark. After washing, 50 µL antibody mix (see Supplementary Table 2) was added and incubated for 30 min at RT in the dark. Data acquisition was performed using the FACSymphony A3 Flow Cytometer (BD Biosciences, NJ, USA). RSV-specific memory CD4^+^ T cells (CD45RA^−^CCR7^-^, CD45RA^+^CCR7^-^, or CD45RA^−^CCR7^+^) were defined through Boolean OR gating. This method allowed us to include all memory CD4^+^ T cells which were positive for at least any two of the activation markers OX40^+^, CD137^+^, CD69^+^ and CD25^+^. This gate is thus a summation of the different double-positive gates, with events occurring in multiple gates only counted once [18]. RSV-specific memory CD8^+^ T cells were defined as CD69^+^CD137^+^. Data were processed using FlowJo software v10.10.0 and OMIQ software (Dotmatics).

Next, we measured cytokines produced in supernatant of PBMCs stimulated with the Select, Fa or Ga peptide pools, with the DMSO condition serving as negative control, using a multiplex human Th cytokine panel kit (LEGENDplex, Biolegend, CA, USA) as described previously [19].

### Statistical analysis

For baseline characteristics of our cohort, continuous data are presented as median and inter-quartile range (IQR) for non-normally distributed variables, and differences between groups were tested using Wilcoxon test. Categorical data are presented as count and percentage, and groups were compared using the Chi-squared test. To compare binding and neutralizing antibody titers between groups and timepoints, Wilcoxon signed rank test with Bonferroni correction for multiple testing was performed, resulting in a significant *p*-value of <0.025. To correlate binding and neutralizing antibody titers, Spearman rank correlation was used to calculate ρ and coefficient.

To obtain the relative frequencies of antigen-specific T cells, the AIM-positive background percentage (as measured in the DMSO condition) was subtracted. Frequencies below 0.01% after adjustment for DMSO were set at 0.01%. The groups were compared using the Kruskal-Wallis test.

The clustering of the cytokine analysis comprised of multiple steps. First, concentrations were adjusted for the DMSO background; negative concentrations were set on 0.01 pg/mL. To avoid false skewing of the analysis, we excluded cytokines for further analysis if more than 50% of observations was negative after adjusting for background, or if more than 20% of observations was under the limit of detection. Next, we calculated the *z*-scores of the log_10_-transformed values. Guided by the agglomerative coefficient of different distance metrics and clustering methods, we continued with the Manhattan distance and Ward method. The optimal number of clusters was chosen based on the largest dissimilarities in the tree diagram. Next, a heatmap was plotted based on the unsupervised hierarchical clustering using the R package pheatmap (v1.0.13). Selected clinical characteristics were compared between the three groups using the Kruskal-Wallis test, and further post-hoc analyses between individual groups using the Wilcoxon test and the Bonferroni correction for multiple testing resulting in a significant *p-*value of <0.017. All analyses were performed in R v4.5.2 and a *p*-value of <0.05 was considered statistically significant, unless stated otherwise. Figures were created in R v4.5.2 and with BioRender Graphing. R scripts are available on request to the corresponding author.

## Results

### Cohort characteristics

We included a total of 64 KTR and 37 controls in our study. The baseline characteristics of the two groups are presented in Table 1. The median time since last transplantation was 6.8 years and the majority of KTR (73.4%) used two immunosuppressant agents. Tacrolimus use (95.3%) and mycophenolate mofetil use (70.3%) were most prevalent in our patient group. There was no significant difference in age or sex between the two groups.

**TABLE 1:** Cohort characteristics.

Characteristic	KTR (n = 64)	Controls (n = 37)	*p*-value
Age (years), median (IQR)	57 (22.75)	66 (18)	0.16^a^
Male sex, n (%)	33 (51.6)	13 (35.1)	0.16^b^
History of dialysis: yes, n (%)	40 (62.5)	​	​
Maintenance IS agent use, n (%) Corticosteroids Tacrolimus Mycophenolate mofetil mTOR inhibitor Azathioprine	24 (37.5) 61 (95.3) 45 (70.3) 3 (4.7) 2 (3.1)	​	​
Number of IS agents, n (%) One Two Three	5 (7.8) 47 (73.4) 12 (18.8)	​	​
Lymphocyte depleting induction, n (%)	5 (7.8)	​	​
First kidney transplant, n (%)	52 (81.3)	​	​
Time since last transplantation (years), median (IQR)	6.8 (9.7)	​	​
Donor type of last transplant, n (%) Living Deceased	42 (65.6) 22 (34.4)	​	​

IS: immunosuppressant; IQR: interquartile range; KTR: kidney transplant recipient.

^a^ *p*-value based on nonparametric test (Wilcoxon).

^b^ *p*-value based on Chi-squared test.

### RSV-specific binding and neutralizing antibodies

We measured pre-F- and Ga-specific IgG and neutralizing antibodies in serum samples collected in March and October 2021; 60 KTR and 28 controls could be measured at both timepoints. Due to limited serum availability, 4 KTR and 7 controls only had a March sample available, and 2 controls only an October sample. Pre-F-specific binding IgG titers were comparable between KTR and controls and were stable over time (Figure 1A). Ga-specific binding IgG titers were comparable between KTR and controls in March, but were significantly higher in KTR than in controls in October (*p* < 0.05; Figure 1B).

**FIGURE 1:**
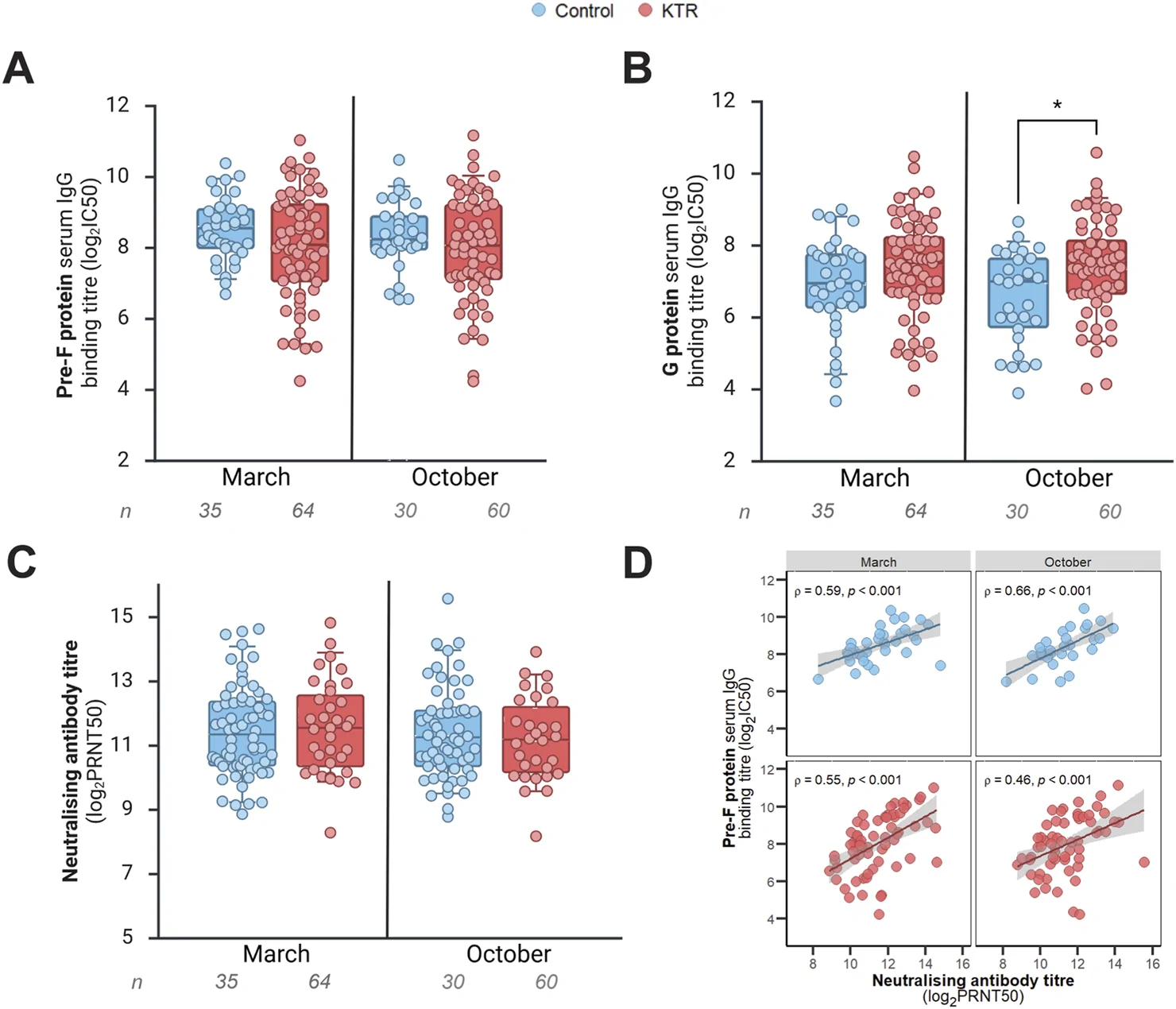
RSV-specific binding and neutralizing antibody titers in March and October 2021 in controls (blue) and KTR (red). Binding antibodies against the **(A)** pre-F and **(B)** Ga protein (*p < 0.05). **(C)** Neutralizing antibodies against rHRSV^A11^EGFP(5). Neutralizing titers are depicted as log_2_ of the calculated PRNT50 value. Horizontal line, box and whiskers indicate medians, interquartile range and limits of 95% CI. **(D)** Correlation between pre-F specific binding antibody titers and neutralizing antibody titers. Spearman rank correlation was used to calculate ρ and coefficient. Regression and 95% confidence band are plotted. Each symbol represents an individual. CI, confidence interval; Ga protein, RSV-A attachment protein of RSV-A; IC50, 50% inhibitory concentration; KTR, kidney transplant recipient; pre-F protein, pre-fusion protein; PRNT50, 50% plaque reduction neutralization titer; rHRSVA11EGFP(5), recombinant RSV containing EGFP; RSV, respiratory syncytial virus.

Neutralizing antibody titers were also comparable between KTR and controls and stable over time (Figure 1C). We subsequently assessed the relationship between binding and neutralizing antibody titers (Figure 1D) and found a moderate but significant correlation in both March and October samples between pre-F-specific binding antibody titers and neutralizing antibody titers for KTR (*rho* = 0.55, *p* < 0.001 and *rho* = 0.46, *p* < 0.001, respectively) and controls (*rho* = 0.59, *p* < 0.001 and *rho* = 0.66, *p* < 0.001, respectively). Similarly, Ga-specific antibody titers correlated with neutralizing antibody titers for both groups and timepoints, albeit less pronounced (*rho* = 0.41, *p* < 0.001 and *rho* = 0.42, *p* < 0.001 for KTR, and *rho* = 0.5, *p* = 0.003 and *rho* = 0.21, *p* = 0.27 for controls; Supplementary Figure S1).

### RSV-specific T cells

To measure RSV-specific T cell responses, we determined the relative frequency of RSV-specific memory T cells in 15 KTR and 14 controls which were randomly selected. Lymphocyte counts were previously determined in whole blood [20] and were significantly lower in KTR compared to controls at both timepoints (*p* < 0.001; Figure 2A). Next, we quantified RSV-specific CD4^+^ and CD8^+^ T cells as relative frequencies of total CD4^+^ or CD8^+^ memory T cells using the gating strategy depicted in Figure 2B. No significant differences in RSV-specific CD4^+^ T cell frequencies were observed for any of the stimuli at either timepoint, except for stimulation with the Fa peptide pool in March, which resulted in higher frequencies observed in KTR (*p* < 0.05; Figure 2C). As expected, the highest frequencies of RSV-specific T cells were detected after stimulation with the Select pool that contained immunogenic peptides, followed by the overlapping F peptide pool from either RSV strain. Similarly, RSV-specific CD8^+^ T cell frequencies were not different between KTR and controls at either timepoint, but were in general lower than RSV-specific CD4^+^ T cell frequencies (Figure 2D).

**FIGURE 2:**
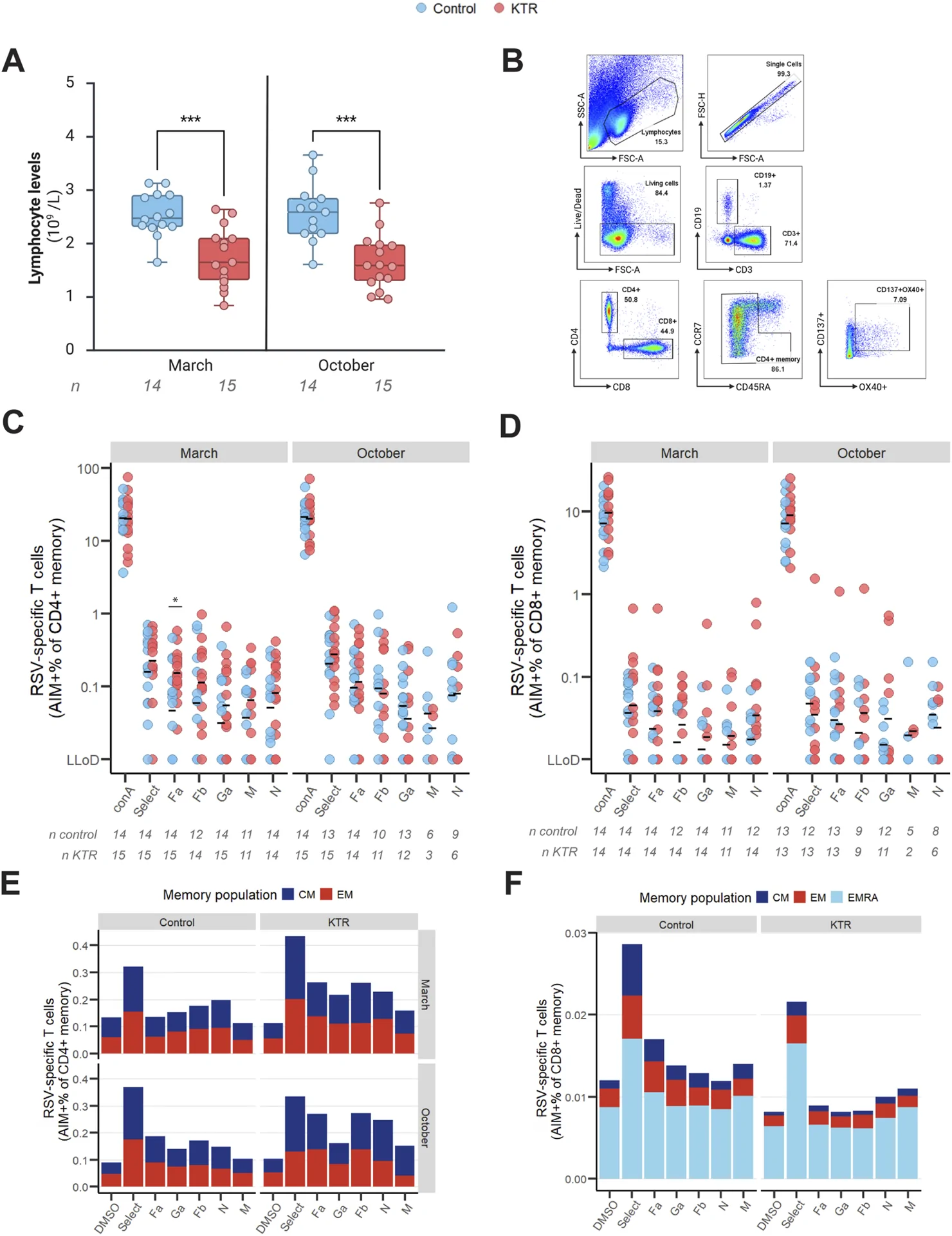
RSV-specific T cells in controls (blue) and KTR (red) in March and October 2021. **(A)** Lymphocyte levels. Horizontal line, box and whiskers indicate medians, interquartile range, and minimum and maximum (***p < 0.001). **(B)** Gating strategy. **(C)** RSV-specific CD4^+^ (*p < 0.05) and **(D)** CD8^+^ T cells as relative frequency of total memory population following stimulation with ConA or different RSV-derived peptide pools. Frequencies are adjusted for DMSO background. Horizontal bars indicate geometric mean and each symbol represents an individual. **(E)** Memory subsets of RSV-specific CD4^+^ T cells as median per cohort per timepoint. **(F)** Memory subsets of RSV-specific CD8^+^ T cells as median per cohort of pooled timepoints. AIM, activation-induced marker; CM, central memory; ConA, Concanavalin A; EM, effector memory; EMRA, effector memory re-expressing CD45RA; Fa, RSV-A fusion protein; Fb, RSV-B fusion protein; Ga, RSV-A attachment protein; LLoD, lower limit of detection; KTR, kidney transplant recipient; RSV, respiratory syncytial virus.

Next, we investigated the memory phenotype of RSV-specific T cells. Within the CD4^+^ T cell compartment, we distinguished central memory (CM, CCR7^+^CD45RA^−^) and effector memory (EM, CCR7^−^CD45RA^−^) populations. To determine the RSV-specific CD4^+^ T cell memory phenotype, we pooled all samples from each group per timepoint and stimulus. Aspecific ConA stimulation indicated a predominance of CM in both groups (Supplementary Figure S2A), whereas RSV-specific T cells showed a balanced distribution of CM and EM populations (Figure 2E). In the CD8^+^ T cell compartment, an additional memory population was defined: terminally differentiated EM re-expressing CD45RA (EMRA, CCR7^−^CD45RA^+^). Due to the low frequency of RSV-specific CD8^+^ T cells, data from both timepoints were pooled per cohort to determine the relative contributions of CM, EM and EMRA populations. ConA stimulation revealed balanced CM, EM, and EMRA proportions in controls but reduced CM and EM proportions in KTR (Supplementary Figure S2B), whereas RSV-specific T cells were predominantly phenotyped as EMRA cells (Figure 2F).

Lastly, we investigated the targets of the RSV-specific CD4^+^ T cell repertoire. To this end, we included all samples that could be tested for all peptide pools, calculated the total RSV-specific CD4^+^ memory relative frequencies across the mutually exclusive stimulations (Fa, Ga, M and N) and determined the proportion attributable to each target. Most individuals exhibited CD4^+^ memory T cell responses against all four proteins, without a clear dominance of any single antigen (Supplementary Figure S2C). KTR showed a distribution comparable to that of controls, but the relative contributions of each protein target varied between individuals.

### Cytokine production profiles

We measured 12 cytokines in the supernatant of PBMCs obtained in March 2021 stimulated with Select, Fa and Ga peptide pools. IFN-γ and IL-2 levels trended higher in KTR compared to controls, though this was not statistically significant (Figures 3A,B). We observed no significant differences in concentrations of other cytokines. Next, we performed unsupervised hierarchical clustering on cytokine signatures after Select peptide pool stimulation, as this resulted overall in the highest cytokine concentrations. Based on our criteria, 7 cytokines were included for further analysis: IL-2, IL-6, IL-10, IFN-γ, TNF-α, IL17-F and IL-22 (Figures 3A,B; Supplementary Figure S3) and 5 cytokines were excluded as per pre-defined exclusion criteria (materials and methods): IL-4, IL-5, IL-9, IL-13 and IL-17A. The clustering analysis resulted in three cytokine clusters with a distinct profile (Figure 3C). KTR and controls were equally distributed throughout these clusters. Cluster 1 was Th_1_-skewed, cluster 2 showed a balanced profile, and cluster 3 was characterized by a non-Th_1_ profile. Of note, individuals in cluster 1 had significantly lower pre-F-specific binding antibody titers compared to cluster 2, indicating a reduced humoral response (*p* < 0.01, Figure 3D). In contrast, individuals in cluster 3 had significantly lower CD4^+^ and CD8^+^ RSV-specific memory T cells compared to cluster 2, indicating a reduced cellular response (*p* < 0.01 and *p* < 0.01, Figures 3E,F). Individuals in cluster 3 were significantly older than individuals in cluster 2 (*p* < 0.01, Figure 3G).

**FIGURE 3:**
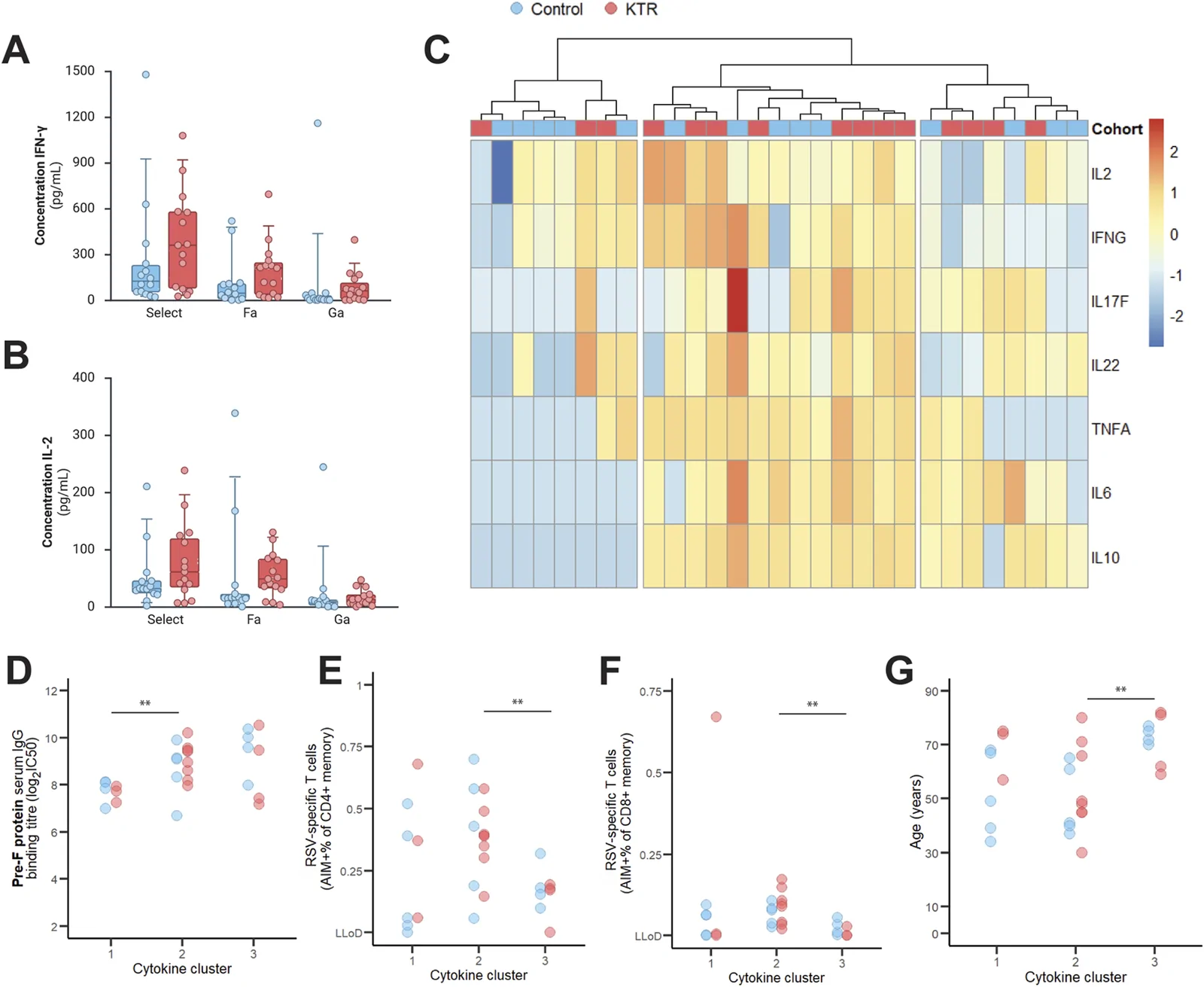
Cytokine profiles after RSV-specific stimulation in controls (blue) and KTR (red) in March 2021. **(A)** IFN-γ and **(B)** IL-2 concentrations following stimulation with Select, Fa or Ga peptide pools after DMSO background subtraction. Horizontal line, box and whiskers indicate medians, interquartile range and limits of 95% CI. **(C)** Heatmap of unsupervised hierarchical cluster analysis using log_10_-transformed *z*-scores of cytokine concentrations after Select pool stimulation. **(D)** Pre-F protein binding antibody titers, **(E)** RSV-specific CD4^+^ and **(F)** CD8^+^ T cells, and **(G)** age per cluster. Symbols represent individuals, **p < 0.01. AIM, activation-induced marker; CI, confidence interval; Fa, RSV-A fusion protein; Fb, RSV-B fusion protein; Ga, RSV-A attachment protein; LLoD, lower limit of detection; KTR, kidney transplant recipient; pre-F protein, pre-fusion protein; RSV, respiratory syncytial virus.

## Discussion

Our study set out to compare RSV-specific humoral and cellular immune responses between KTR and controls. We demonstrated that pre-F- and G-specific binding antibody titers and neutralizing antibodies were comparable between KTR and controls. While total lymphocyte levels were significantly lower in KTR, the relative frequency of RSV-specific CD4^+^ and CD8^+^ T cells was comparable to controls. Finally, we distinguished three cytokine clusters after RSV-specific stimulation of PBMCs, each associated with distinct immunological features: KTR and controls were equally dispersed throughout. Combined, our data suggests that RSV-specific humoral and cellular immune responses are maintained after transplantation.

The comparable antibody titers between KTR and controls indicate that baseline RSV-specific humoral responses, initially induced prior to transplantation, are minimally affected by the immunocompromised state of KTR. This is in line with previous work that measured pan-RSV-specific antibody levels [21]. Ga-specific binding antibody titers in October samples were significantly higher in KTR compared to controls. However, the biological significance of this finding is likely limited since neutralizing antibody titers at that same timepoint showed no significantly difference. Because of the contribution of pre-F-specific antibodies to neutralization, and the fact that pre-F is the basis of monoclonal antibodies and vaccines, we specifically measured antibodies binding this target [12]. As expected, pre-F-specific binding antibody titers correlated to neutralizing antibody titers.

We found that the relative frequency of RSV-specific T cells in the CD4^+^ or CD8^+^ memory compartment was comparable between KTR and controls. KTR have significantly less lymphocytes, though still within the normal range (0.5–5.0 * 10^9^/L). Although lymphocyte numbers are reduced in KTR due to immunosuppressant use, they retain a similar proportion of RSV-specific T cells. The immunosuppressants taken by KTR similarly target all lymphocytes, not immune cells with a certain specificity, which can explain our findings. The similar percentage of RSV-specific T cells in KTR compared to controls was also reported by another study and suggests that pre-existing virus-specific immune responses are not severely affected by (long-term) immunosuppressant use [21]*.* Moreover, the memory phenotype of RSV-specific T cells was similar in KTR and controls, independent of the peptide pool stimulation. The exact functions of these populations within the RSV-specific immune response are not well described, though RSV vaccination was shown to primarily boost the CM population [22]. Furthermore, we demonstrate that KTR have RSV-specific T cells targeting different viral proteins, similar to controls. This further indicates that the comparable RSV-specific T cell frequencies in KTR are not driven by a monoclonal response but are the result of a diverse RSV-specific T cell repertoire. However, since our assays were performed *ex vivo* in the absence of immunosuppression, activation potential may be overestimated.

Virus-specific cytokine production also did not differ significantly between KTR and controls. To achieve viral clearance without excessive immunopathology after RSV infection, a balanced cytokine production by both CD4^+^ and CD8^+^ T cells is crucial [23, 24]. Our cluster analysis revealed three distinct cytokine profiles, with KTR and controls dispersed throughout, further supporting that RSV-specific cellular responses are comparable between KTR and controls. Noteworthy is that RSV-specific immune responses did differ between the three clusters. Cluster 1, characterized by production of predominantly Th_1_ cytokines, showed an impaired humoral response compared to cluster 2, characterized by a balanced cytokine profile. These results are in accordance with a recent study on COVID-19-specific immune responses in KTR [19]. In contrast, cluster 3, characterized by production of cytokines other than Th_1_, showed an impaired cellular response and had significantly older individuals compared to cluster 2. These two findings are unlikely to be confounders, as previous studies also show that older individuals do not have less RSV-specific T cells [25]. Rather, these are independently associated with a non-Th_1_ cytokine profile which warrants further research for clinical implications. Additionally, we compared G-specific and neutralizing antibody titers, T cell memory phenotypes and lymphocyte levels between clusters, but found no differences. Furthermore, no differences in cohort characteristics were observed, although this may be restricted by the limited number of KTR included in the PBMC analysis.

A strength of this study is that we provide a comprehensive overview of the RSV-specific immune response. Especially in immunocompromised patients, assessment of virus-specific immune responses warrants a holistic approach, as immunosuppressive regimens often target different arms of the adaptive immune system. Thus, a focus on antibodies alone is undesirable. This is further supported by studies that found vaccine-induced T cell responses to SARS-CoV-2 in KTR without serum neutralizing activity [26, 27]. Moreover, though we cannot rule out that RSV infections occurred during the study period, these samples provided novel insights. The social distancing measures put in place due to the COVID-19 pandemic led to a prolonged period of limited respiratory virus circulation, permitting a unique baseline perspective on RSV-specific immunity. As no significant differences were observed between timepoints, it is likely that RSV infections were rare, if not absent, in our cohort. This likelihood is further strengthened by the observations that, despite the lifting of social distancing measures in the general Dutch population in the summer of 2021, our cohort was known to show high adherence due to their risk group status. Additionally, since controls in the RECOVAC consortium were partners, siblings or household members of the included kidney patients [20], our control group was also likely to show persistent adherence to social distancing measures.

We also note several limitations. Our findings are immune-based, and do not directly translate to protection against (severe) disease after RSV infection, which is limited by the lack of an established correlate of protection for RSV. Additionally, we used PBMCs to investigate the T cell compartment, but these do not necessarily provide a view of the T cell populations in secondary lymphoid tissues or the site of infection. The importance of tissue-resident memory (TRM) cells in protection against RSV disease has been previously described [28]. Moreover, several studies indicate that levels of circulating RSV-specific T cells do not necessarily reflect levels of TRM [28, 29]. Furthermore, our sample size is limited, which hinders generalization of the results and pre-transplant samples were not available, which limits determining the persistence of pre-existing immunity within individual KTR. Lastly, our findings do not include recall responses after vaccination or infection after transplantation. However, recent studies on RSV vaccination in solid organ transplant recipients demonstrate that pre-existing RSV-specific immunity can increase in response to vaccination in a similar fashion to controls, albeit slower [21, 22, 30].

To conclude, our findings suggest that pre-existing RSV-specific immune responses are maintained after kidney transplantation, despite long-term use of immunosuppressive medication. However, the extent to which this can be translated to protection against (severe) disease following RSV infection remains unclear, since KTR have less lymphocytes than controls. Nonetheless, our study highlights the potential of vaccination targeting viruses initially encountered before transplantation, as boosting pre-existing immunity is likely more efficient than *de novo* immune induction.

## Data Availability

The raw data supporting the conclusions of this article will be made available by the authors, without undue reservation.
