# Supplementary material for: RSV-specific humoral and cellular immune responses in kidney transplant recipients
Source: Transpl Int. 2026 Oct 30;39:17558. doi: 10.3389/ti.2026.17558 (PMC13760235; doi:10.3389/ti.2026.17558)
Supplement: Supplementary file 1 [file DataSheet1.pdf]

Supplemental Table 1: Peptide pools

Full name	Shortened name	Manufacturer
PepMix Pan-RSV Select	Select	JPT Technologies
PepMix HRSVA (Fusion Glycoprotein F0)	Fa	JPT Technologies
PepMix HRSVB (Fusion Glycoprotein F0)	Fb	JPT Technologies
PepMix HRSVA (Major Surface Glycoprotein G)	Ga	JPT Technologies
PepMix HRSVA (Matrix Protein)	M	JPT Technologies
PepMix HRSVB (Nucleocapsid Protein N)	N	JPT Technologies

Supplemental Table 2: Antibody mix

Marker	Dye	Clone	Manufacturer	Cat	Lot
FVS780 (Live/Dead)	APC-Cy7	-	BD	565388	4002454
CD3	AF700	HIT3a	BioLegend	300324	B425949
CD4	PerCP-Cy5.5	RPA-T4	BD	560650	4120178
CD8	BV510	RPA-T8	BioLegend	301048	B438561
CD19	PE-Cy7	SJ25C1	BioLegend	363011	B411739
CD56	BV785	5.1H11	BioLegend	362550	B427457
CD14	BV605	63D3	BioLegend	367126	B427075
CCR7	PE	G043H7	BioLegend	353204	B427560
CD45RA	BV650	HI100	BioLegend	304135	B429182
CD69	FITC	FN50	BioLegend	310904	B417168
CD25	BV711	MA251	BioLegend	356138	B444877
CD137	APC	4B4-1	BD	550890	4130502
OX40	BV421	Ber-ACT35	BioLegend	350014	B389965
Brilliant stain buffer	-	-	BD Horizon	566385	4214476

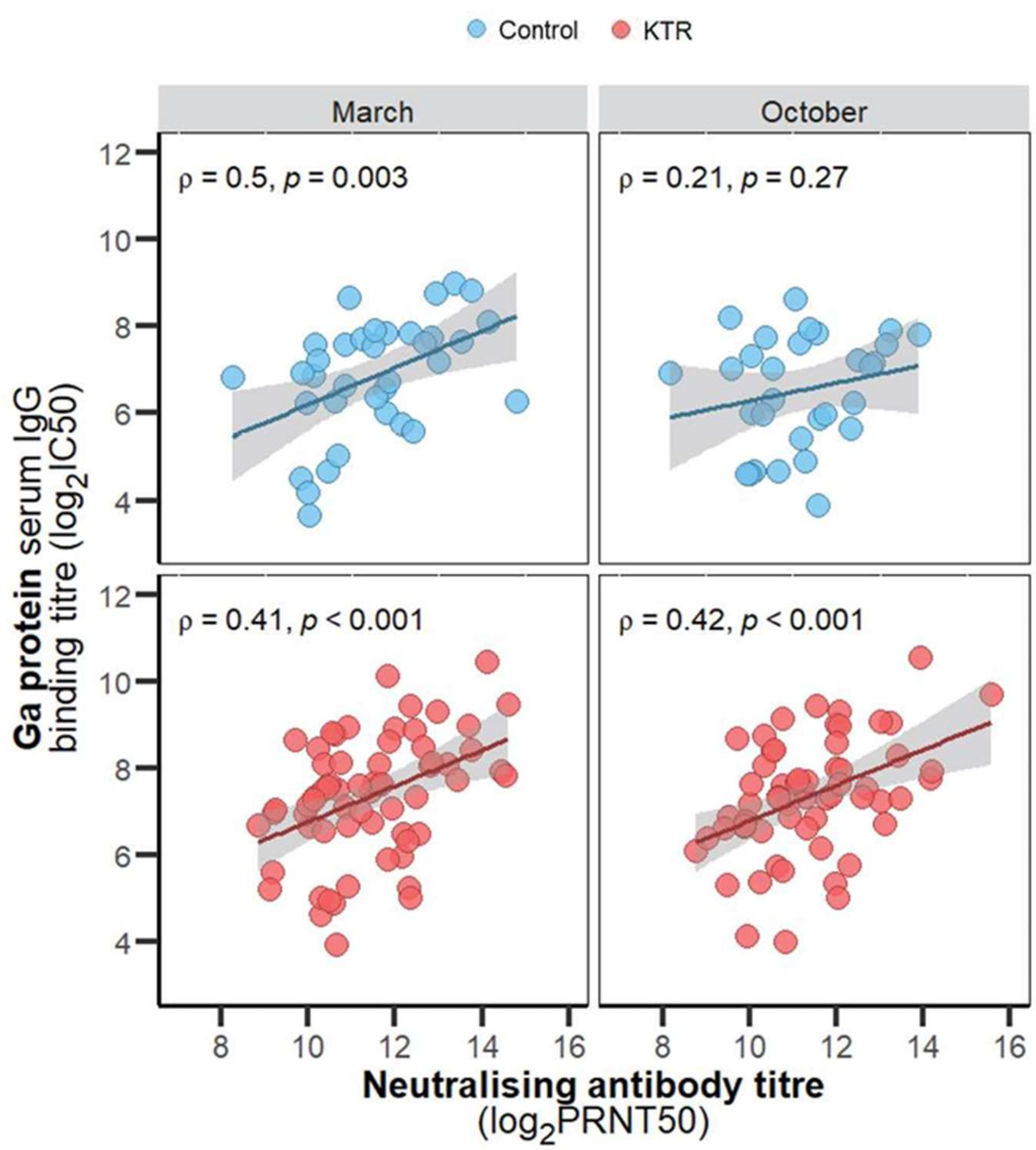

Supplemental Figure 1: Correlation between Ga-specific binding and neutralising antibody titres in KTR (red) and controls (blue) in March and October 2021.

Spearman rank correlation was used to calculate ρ and coefficient. Regression and 95% confidence band are plotted. Each symbol represents an individual. CI, confidence interval; Ga protein, RSV-A attachment protein; IC50: 50% inhibitory concentration; KTR: kidney transplant recipient; PRNT50: 50% plaque reduction neutralisation titre.

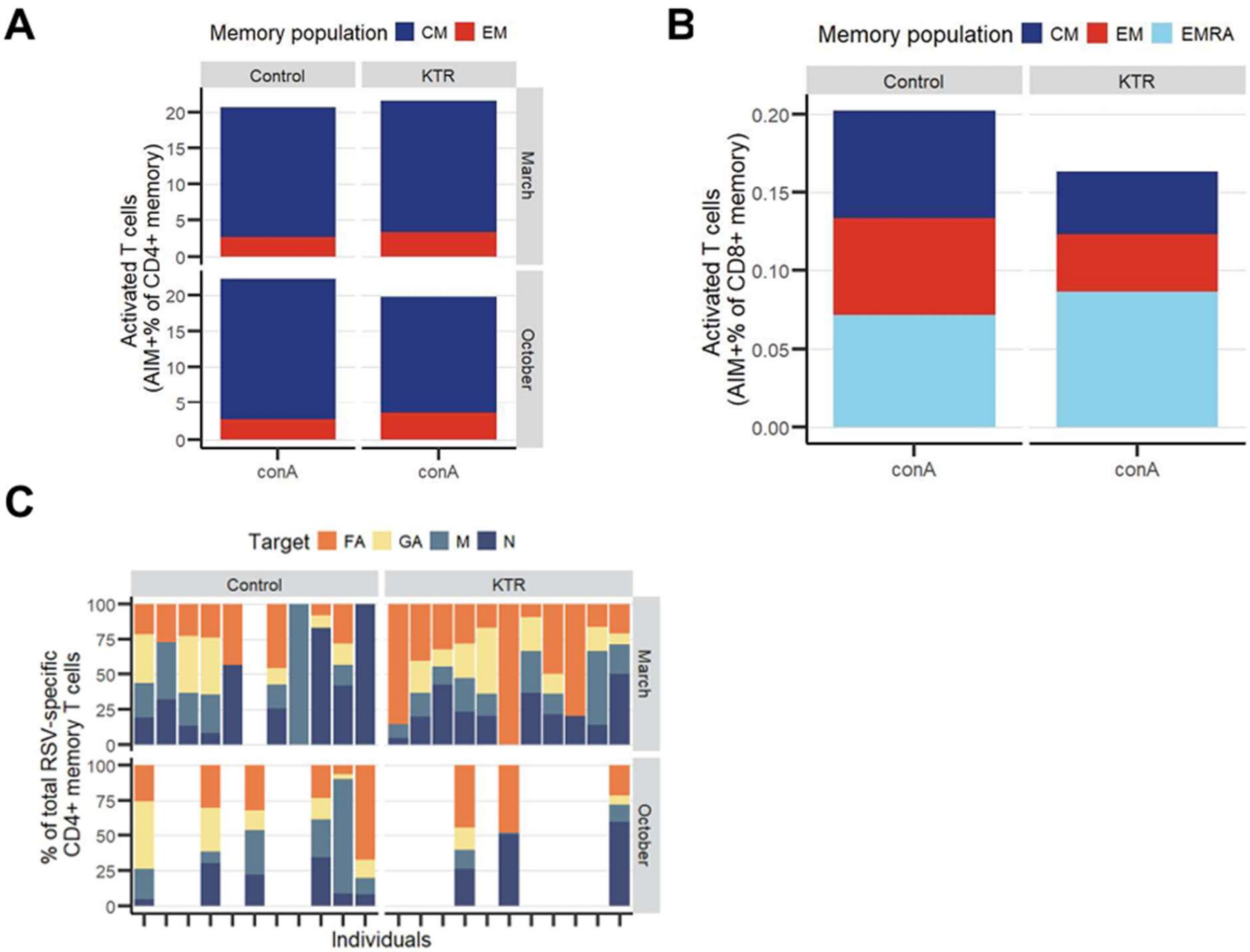

Supplemental Figure 2: T cells in controls and KTR

A) Memory subsets of activated CD4⁺ T cells following conA stimulation as median per cohort per timepoint. B) Memory subsets of activated CD8⁺ T cells following conA stimulation as median per cohort of pooled timepoints. C) RSV-specific CD4⁺ memory T cells after stimulation with Fa, Ga, M or N peptide pools, as proportion of total RSV-specific CD4+ memory T cells detected. Each column represents an individual. AIM, activation-induced marker; CM, central memory; conA, Concanavalin A; EM, effector memory; EMRA, effector memory re-expressing CD45RA; Fa, RSV-A fusion protein; Ga, RSV-A attachment protein; KTR, kidney transplant recipient; RSV, respiratory syncytial virus.

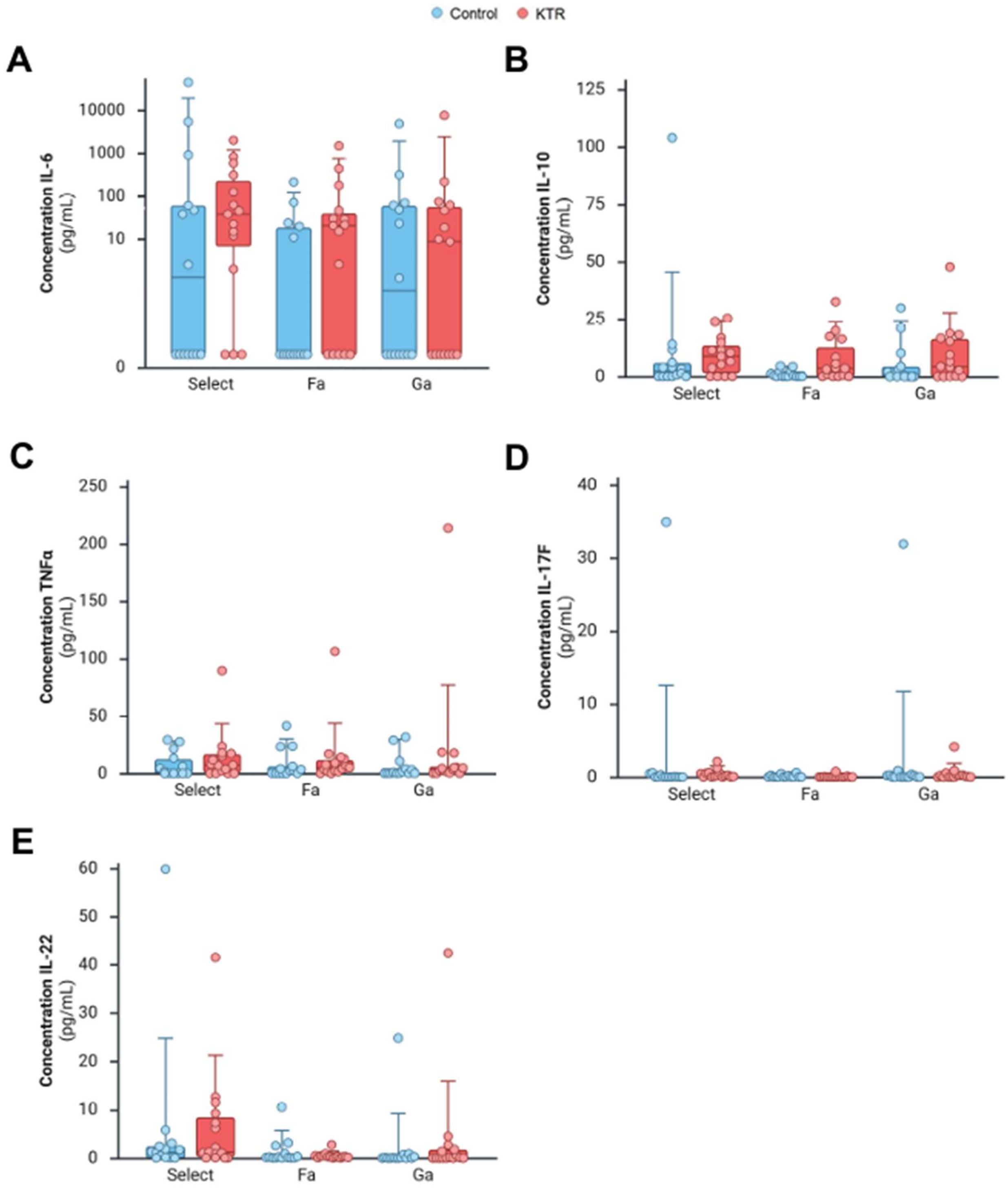

Supplemental Figure 3: Cytokines included in clustering analysis

A) IL-6, B) IL-10, C) TNFα, D) IL-17F and E) IL-22 concentrations following stimulation with Select, Fa or Ga peptide pools after DMSO background subtraction. Horizontal line,

box and whiskers indicate medians, interquartile range and limits of 95% CI. CI, confidence interval; Fa, RSV-A fusion protein; Ga, RSV-A attachment protein; KTR, kidney transplant recipient.
